# CBC‑derived inflammatory indices for rheumatoid arthritis diagnosis and activity assessment: differential performance by serostatus

**DOI:** 10.3389/fimmu.2026.1740898

**Published:** 2026-01-21

**Authors:** Jing Zhang, Yuwei Wang, Weiduo Nie, Qianpeng Li, Sheng-Guang Li, Di Jin

**Affiliations:** 1 Department of Rheumatology and Immunology, Peking University International Hospital, Beijing, China; 2 Department of Emergency Internal Medicine, Weifang People’s Hospital, Weifang, Shandong, China; 3 Dongfang Hospital, Beijing University of Chinese Medicine, Beijing, China; 4 Department of Hematology, Weifang People’s Hospital, Shandong Second Medical University, Weifang, Shandong, China; 5 Department of Rheumatology, Weifang People’s Hospital, Weifang, Shandong, China

**Keywords:** complete blood cell count (CBC)-derived inflammatory indicator, disease activity, neutrophil-to-lymphocyte ratio, rheumatoid arthritis, seronegative rheumatoid arthritis, systemic immune-inflammation index

## Abstract

**Background:**

Rheumatoid arthritis (RA) is an autoimmune disease for which better biomarkers are needed, especially in seronegative cases. This study evaluates complete blood count (CBC)-derived inflammatory indices – neutrophil-to-lymphocyte ratio (NLR), platelet-to-lymphocyte ratio (PLR), monocyte-to-lymphocyte ratio (MLR), systemic immune-inflammation index (SII), and systemic inflammation response index (SIRI) – for RA diagnosis and disease activity assessment, with comparisons between seropositive and seronegative RA.

**Methods:**

We conducted a retrospective case–control study of 230 RA patients and 115 age- and sex-matched healthy controls. CBC-derived indices were calculated from routine blood counts. Diagnostic performance was evaluated using receiver operating characteristic (ROC) curves (area under the curve, AUC) for RA versus controls overall and stratified by serostatus. Associations with disease activity (DAS28-CRP, SDAI, CDAI) were assessed via correlations and ROC analysis for active (moderate/high) versus inactive (remission/low) RA.

**Results:**

All five indices were significantly elevated in RA patients compared to controls (all *P* < 0.001). MLR showed the highest diagnostic accuracy (AUC = 0.771), followed by SIRI (0.72) and PLR (0.70); NLR and SII were more modest (≈0.69–0.68). In seronegative RA, diagnostic discrimination declined (best AUC = 0.707 for MLR; SII and SIRI AUCs ~0.56–0.59). NLR, SII, and SIRI correlated moderately with CRP, ESR, and composite scores (Spearman ρ ~0.3–0.4, *P* < 0.001), and were higher in active RA (DAS28-CRP AUCs 0.668–0.700). SII and SIRI provided the top discrimination of active disease (AUC ~0.70). PLR showed minimal correlation with activity and no significant difference between active and inactive RA.

**Conclusion:**

CBC-derived inflammatory indices are elevated in RA and reflect systemic inflammation. MLR is the most robust index for distinguishing RA from healthy individuals, while SII, SIRI, and NLR are useful for gauging disease activity. In seronegative RA, diagnostic performance was attenuated overall, with MLR retaining fair discrimination while SII/SIRI/NLR showed limited utility.

## 1 Introduction

Rheumatoid arthritis (RA) is a chronic autoimmune disease characterized by persistent synovitis that can lead to joint destruction, disability, and extra-articular complications (1). Early diagnosis and accurate assessment of disease activity are critical to preventing irreversible damage and improving long-term outcomes (2). However, diagnostic challenges remain, particularly in early or seronegative RA, where patients lack rheumatoid factor (RF) and anti-citrullinated protein antibodies (ACPA). Seronegative RA accounts for 20–30% of cases and often experiences delayed recognition and treatment initiation, resulting in worse prognosis (3).

Beyond conventional biomarkers such as C-reactive protein (CRP) and erythrocyte sedimentation rate (ESR) (4), several indices derived from routine complete blood counts (CBC) have emerged as potential markers of systemic inflammation. These include the neutrophil-to-lymphocyte ratio (NLR), platelet-to-lymphocyte ratio (PLR), monocyte-to-lymphocyte ratio (MLR), the systemic immune-inflammation index (SII), and the systemic inflammation response index (SIRI). CBC-derived indices are inexpensive, widely available, and easily calculated, making them attractive for use in daily clinical practice (5, 6).

Growing evidence suggests that these indices reflect disease activity and outcomes in autoimmune diseases. In anti-neutrophil cytoplasmic antibody-associated vasculitis (AAV), NLR, PLR, MLR, SII, and SIRI are significantly elevated compared to healthy controls and correlate with disease activity and prognosis (7–9). Similar associations have been reported in systemic lupus erythematosus (SLE) and idiopathic inflammatory myopathies (10, 11). In RA, small studies have indicated that NLR and PLR are higher in patients than in controls and associated with disease activity scores, but the overall evidence remains limited (12–15).

Importantly, most prior RA studies did not stratify patients by serostatus, despite the clinical and immunological heterogeneity between seropositive and seronegative RA. Moreover, comparative evaluation of all five indices in a single cohort has been lacking, and their ability to predict active disease remains uncertain (16).

Therefore, the present study aimed to systematically evaluate the diagnostic performance and activity-related utility of NLR, PLR, MLR, SII, and SIRI in a large RA cohort, with a specific focus on differential performance between seropositive and seronegative patients.

## 2 Methods

### 2.1 Study design and setting

This study was a retrospective case–control analysis conducted at a single center (Department of Rheumatology, Weifang People’s Hospital, Weifang, Shandong, China) between January 1, 2020, and June 30, 2025. All procedures were approved by the institutional ethics committee, and the requirement for informed consent was waived due to the study’s retrospective nature.

### 2.2 Participants

Eligible participants were adult RA patients (age ≥ 18 years) who met the 2010 American College of Rheumatology/European League Against Rheumatism (ACR/EULAR) classification criteria for RA (17). We excluded patients with coexisting malignancies, active infections, pregnancy, major organ failure (e.g., end-stage cardiac, hepatic, or renal disease), or any overlap syndrome with another rheumatologic autoimmune disease. Healthy controls were recruited from routine health check-up attendees at the same hospital, matched for age and sex distribution to the RA group, with no known chronic illnesses or autoimmune disorders.

Within the RA cohort, we further stratified patients by serostatus: seropositive RA was defined as those with positive RF and/or ACPA, while seronegative RA included patients negative for both RF and ACPA.

### 2.3 Clinical and laboratory data

For all included subjects, we collected comprehensive clinical and laboratory information from medical records. Demographic variables (age and sex) and serologic markers (RF and ACPA status) were recorded, as were standard inflammatory markers (CRP and ESR). Complete blood count (CBC) parameters were obtained from the same visit, including white blood cell count, differential counts (neutrophils, monocytes, lymphocytes), platelet count, and hemoglobin level.

For patients with multiple visits during the study period, one index visit per patient was selected; the index visit was defined as the earliest eligible encounter with available CBC and disease activity data to ensure one observation per patient.

All laboratory measurements were performed in the hospital’s central laboratory using automated analyzers and standard methods (e.g., immunoturbidimetry for CRP, Westergren method for ESR), ensuring consistency throughout the study period. From the CBC data, we calculated five inflammation-related indices for each individual: Neutrophil–lymphocyte ratio (NLR =neutrophil count/lymphocyte count), Platelet–lymphocyte ratio (PLR= platelet count/lymphocyte count),Monocyte–lymphocyte ratio (MLR= monocyte count/lymphocyte count),Systemic immune-inflammation index (SII = (platelet count × neutrophil count)/lymphocyte count) (18), Systemic inflammation response index (SIRI = (neutrophil count × monocyte count)/lymphocyte count) (19). These indices were chosen based on prior evidence suggesting their utility as inflammation markers in RA populations (14, 20). All clinical and laboratory data were cross verified by two researchers before analysis to ensure accuracy.

### 2.4 Disease activity classification

Disease activity was assessed at the index visit using DAS28-CRP/DAS28-ESR, SDAI, and CDAI, based on clinical assessments and laboratory results recorded at that same encounter. Patients’ tender and swollen joint counts, global assessments, and CRP were documented for index calculations. Disease activity levels were defined according to standard thresholds. DAS28-CRP and DAS28-ESR: remission if DAS28 ≤ 2.6; low activity if DAS28 > 2.6 and ≤ 3.2; moderate if DAS28 > 3.2 and ≤ 5.1; high activity if DAS28 > 5.1 (21). SDAI: remission if ≤ 3.3; low activity if ≤ 11; moderate if ≤ 26; high activity if > 26 (22). CDAI: remission if ≤ 2.8; low activity if ≤ 10; moderate if ≤ 22; high activity if > 22 (22).

For certain analyses, we dichotomized disease activity into “active RA” (moderate or high disease activity) versus “non-active” (remission or low disease activity). Specifically, active RA was defined as having at least moderate activity by composite indices (e.g., DAS28-CRP > 3.2 or SDAI > 11), while remission or low disease activity were considered non-active.

### 2.5 Statistical analysis

All statistical analyses were performed using Python and R software (v4.2.0). Continuous variables were first tested for normality using the Shapiro–Wilk test (23). Data following a normal distribution are presented as mean ± standard deviation and were compared between groups using the independent-samples *t*-test. Non-normally distributed data are presented as median with interquartile range (IQR) and were compared using the Mann–Whitney U test (for two-group comparisons). Categorical variables (e.g., sex, seropositivity) were summarized as counts and percentages, and compared using the chi-square (χ²) test (or Fisher’s exact test if an expected cell count was < 5).

We evaluated the associations between inflammatory indices and disease activity using Spearman’s rank correlation coefficient, given the non-parametric nature of most variables. To assess the diagnostic and predictive performance of the CBC-derived indices, we carried out ROC curve analyses. ROC curves were constructed to distinguish RA patients from healthy controls (diagnostic performance of each index) and to distinguish active RA (moderate/high disease activity) from inactive disease (remission/low activity) within the RA cohort. The area under the ROC curve (AUC) was calculated for each index along with its 95% confidence interval, and comparisons of AUCs were made when necessary. For each ROC analysis, the optimal cutoff value for the index was determined by maximizing the Youden index (sensitivity + specificity – 1) (24), and the corresponding sensitivity and specificity at that cutoff were recorded.

Ninety-five percent confidence intervals (95% CIs) for sensitivity and specificity were calculated using binomial proportion methods, and 95% CIs for the Youden-optimized cut-off values were estimated using nonparametric bootstrap resampling (2,000 replicates).

All significance tests were two-tailed, and a *P*-value < 0.05 was considered statistically significant for all comparisons. Data handling and reporting conformed to STROBE guidelines for observational studies (25).

### 2.6 Ethical considerations

This study was conducted in accordance with the ethical principles of the Declaration of Helsinki. The study protocol was reviewed and approved by the Ethics Committee of Weifang People’s Hospital (Weifang, China) (approval number: [KYLL20251029-2]).

## 3 Results

### 3.1 Participant characteristics and baseline comparison

We included 230 patients with RA and 115 age- and sex-matched healthy controls. Groups were comparable for age (60.9 ± 10.8 vs. 59.7 ± 6.7 years, *P* = 0.67) and sex (female 90.4% vs. 87.8%, *P* = 0.58). The RA cohort had a median disease duration of 36 months (IQR 12–60), and 74.8% were seropositive for RF and/or ACPA. Compared with controls, RA patients had significantly higher neutrophil, and monocyte counts, and lower lymphocyte counts and hemoglobin (all *P* < 0.001), whereas total leukocyte and platelet counts were similar between groups. At baseline, median DAS28-CRP was 3.31; 56.5% of patients had moderate or high disease activity by DAS28-CRP, and 32.6% by SDAI. Full details are provided in **Table 1**.

**Table 1:** Baseline characteristics of RA patients and healthy controls. Values are mean ± SD or n (%).

Variable	RA patients (n = 230)	Healthy controls (n = 115)	*P*-value
Age (years)	60.9 ± 10.8	59.7 ± 6.7	0.67
Female, n (%)	208 (90.4%)	101 (87.8%)	0.58
Disease duration (months) †	47.5 ± 50.0	–	–
White blood cell count (×10^9/L)	6.49 ± 4.73	5.93 ± 1.33	0.52
Neutrophil count (×10^9/L)	4.01 ± 1.84	3.47 ± 1.04	0.025
Lymphocyte count (×10^9/L)	1.64 ± 0.66	1.94 ± 0.46	< 0.001
Monocyte count (×10^9/L)	0.43 ± 0.18	0.37 ± 0.25	< 0.001
Platelet count (×10^9/L)	249.4 ± 69.8	234.7 ± 47.7	0.19
Hemoglobin (g/L)	132.1 ± 33.2	142.9 ± 12.0	< 0.001
NLR	2.83 ± 2.00	1.87 ± 0.66	< 0.001
PLR	175.2 ± 82.9	126.4 ± 34.6	< 0.001
MLR	0.298 ± 0.175	0.190 ± 0.095	< 0.001
SII	711.4 ± 535.9	438.8 ± 178.0	< 0.001
SIRI	1.24 ± 0.98	0.67 ± 0.40	< 0.001

†Disease duration available for RA patients only (mean ± SD 47.5 ± 50.0 months, median 36 months).

Statistical comparisons by *t*-test or χ² test as appropriate.

### 3.2 Group differences in CBC-derived inflammatory indices

**Figure 1:**
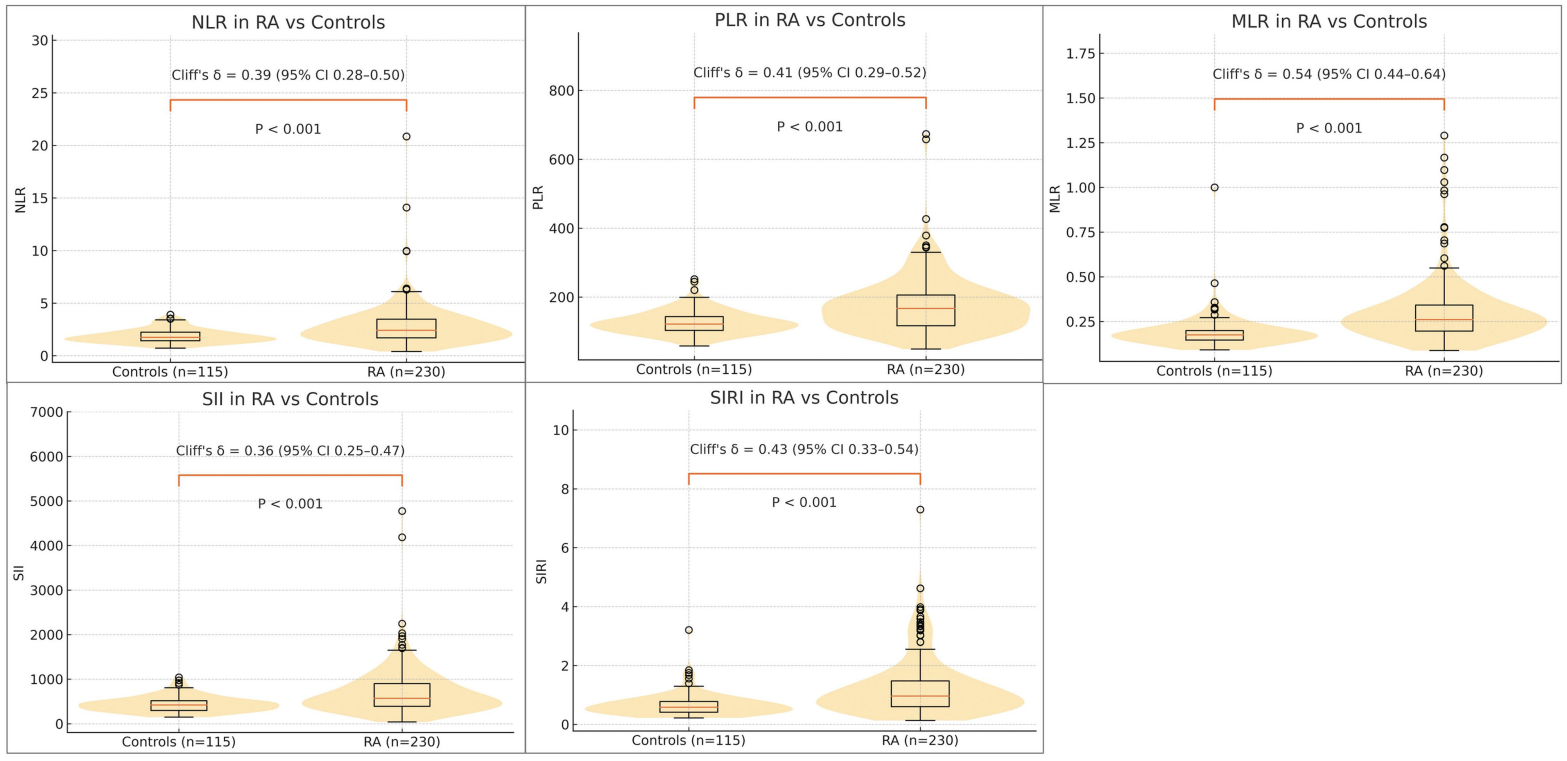
Distributions of CBC-derived inflammatory indices in rheumatoid arthritis (RA) versus healthy controls. NLR, MLR, PLR, SII, SIRI. Each panel overlays a violin with a boxplot (median, IQR, whiskers) to compare RA (n = 230) with controls (n = 115). All five indices are elevated in RA (two-sided Mann–Whitney U; all *P* < 0.001). Effect sizes (Cliff’s δ with 95% CI) are annotated; MLR and SIRI show the largest separation, whereas NLR/PLR/SII display broader but still right-shifted distributions.

All five indices were markedly higher in RA than in controls (all *P* < 0.001; **Table 1**). Distribution plots showed the smallest overlap between RA and controls for MLR and SIRI, whereas NLR, PLR, and SII displayed wider ranges (**Figure 1**).

### 3.3 Diagnostic performance in the overall cohort

Receiver operating characteristic analyses demonstrated statistically significant discrimination of RA vs. controls for all indices (all P < 0.001; AUCs 0.68–0.77). MLR provided the highest diagnostic accuracy (AUC = 0.771, 95% CI 0.720–0.820), slightly outperforming SIRI (AUC = 0.72) and PLR (AUC = 0.70), with NLR and SII being more modest (AUCs ~0.69 and 0.68, respectively). Detailed cut-offs and metrics (including 95% CIs) are summarized in **Table 2**.

**Table 2:** Diagnostic performance of CBC-derived inflammatory indices for RA versus healthy controls (overall and by serostatus).

Comparison	Marker	Cut-off (95% CI)	AUC (95% CI)	Sensitivity % (95% CI)	Specificity % (95% CI)	P-value
All RA vs Control	NLR	2.29 (1.90–2.79)	0.69 (0.64–0.75)	55.22 (48.8–61.5)	79.13 (70.8–85.6)	<0.001
	PLR	165.75 (142.50–176.25)	0.70 (0.65–0.76)	51.74 (45.3–58.1)	88.70 (81.6–93.3)	<0.001
	MLR	0.21 (0.208–0.223)	0.77 (0.72–0.82)	72.61 (66.5–78.0)	80.87 (72.7–87.0)	<0.001
	SII	565.63 (515.82–803.08)	0.68 (0.63–0.74)	51.74 (45.3–58.1)	80.87 (72.7–87.0)	<0.001
	SIRI	0.86 (0.635–1.079)	0.72 (0.66–0.77)	56.96 (50.5–63.2)	82.61 (74.7–88.5)	<0.001
SPRA vs Control	NLR	2.29 (1.88–2.79)	0.73 (0.67–0.78)	58.72 (51.3–65.8)	79.13 (70.8–85.6)	<0.001
	PLR	166.13 (139.22–176.25)	0.73 (0.67–0.78)	54.65 (47.2–61.9)	88.70 (81.6–93.3)	<0.001
	MLR	0.21 (0.208–0.224)	0.79 (0.74–0.84)	77.33 (70.5–82.9)	79.13 (70.8–85.6)	<0.001
	SII	567.10 (515.82–824.41)	0.72 (0.66–0.78)	55.23 (47.8–62.5)	81.74 (73.7–87.7)	<0.001
	SIRI	0.86 (0.761–1.073)	0.76 (0.70–0.81)	62.21 (54.8–69.1)	82.61 (74.7–88.5)	<0.001
SNRA vs Control	NLR	2.55 (1.92–3.60)	0.60 (0.50–0.70)	43.10 (31.2–55.9)	85.22 (77.6–90.6)	0.01
	PLR	175.76 (143.33–187.14)	0.64 (0.54–0.73)	39.66 (28.1–52.5)	92.17 (85.8–95.8)	<0.01
	MLR	0.21 (0.196–0.220)	0.71 (0.62–0.80)	65.52 (52.7–76.4)	80.00 (71.8–86.3)	<0.001
	SII	542.86 (542.86–854.87)	0.56 (0.46–0.66)	44.83 (32.7–57.5)	77.39 (68.9–84.1)	0.09
	SIRI	1.07 (0.601–1.176)	0.59 (0.49–0.69)	32.76 (22.1–45.6)	93.04 (86.9–96.4)	0.02

Receiver operating characteristic (ROC) curve analyses were performed to evaluate the diagnostic performance of CBC-derived inflammatory indices for rheumatoid arthritis (RA) compared with healthy controls, in the overall cohort and stratified by serostatus. Optimal cut-off values were determined by maximizing the Youden index. Area under the curve (AUC) values are presented with 95% confidence intervals (CIs). Sensitivity and specificity are shown with corresponding 95% CIs. The 95% CIs for cut-off values were estimated using nonparametric bootstrap resampling (2,000 replicates). *SPRA*, seropositive RA; *SNRA*, seronegative RA.

### 3.4 Serostatus-stratified diagnostic performance

**Figure 2:**
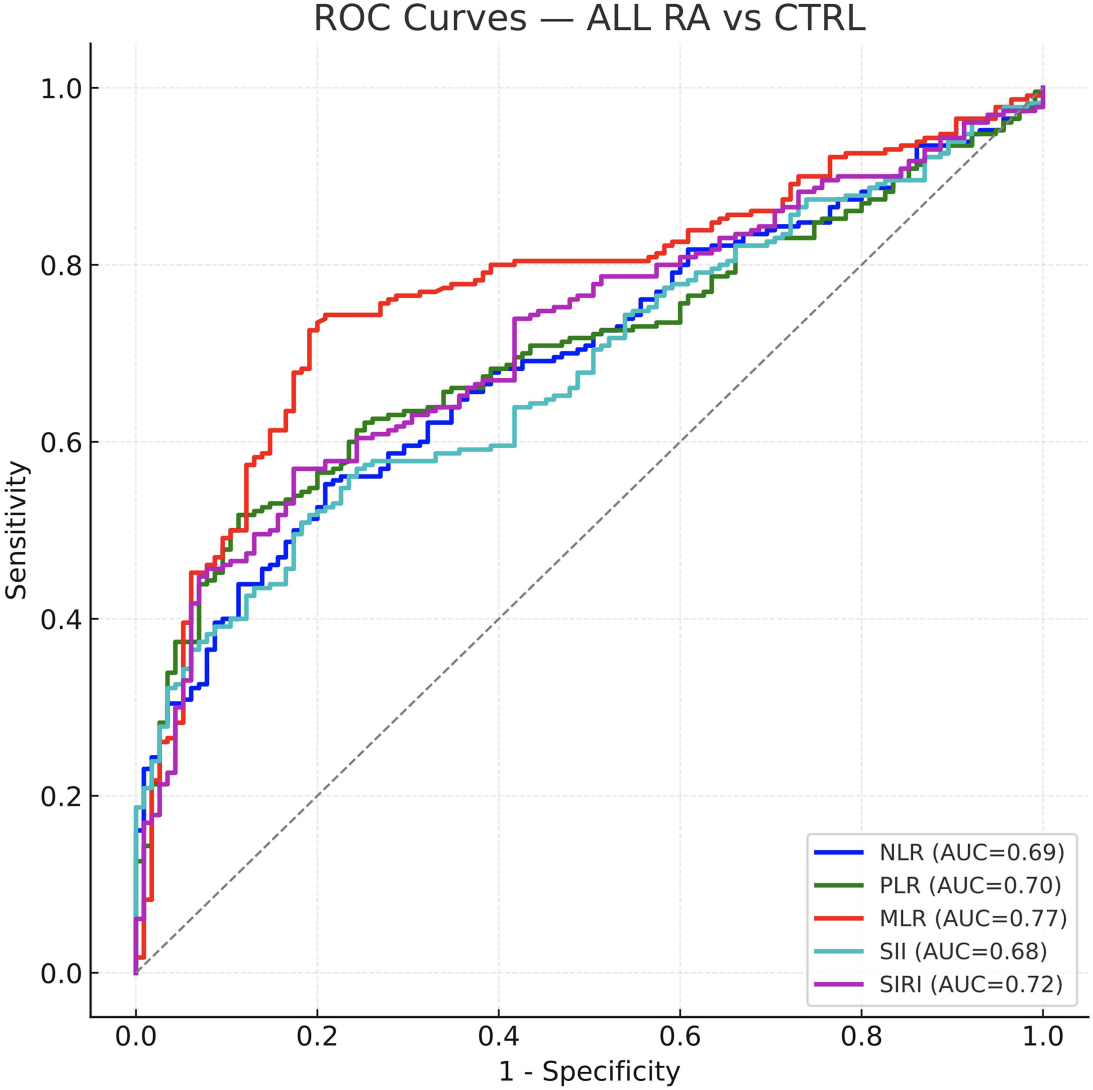
ROC curves of inflammatory indices for diagnosing RA versus healthy controls (overall cohort). NLR, MLR, PLR, SII, SIRI, all indices discriminate RA from controls (AUC ≈ 0.68–0.77; *P* < 0.001). MLR yields the highest AUC (~0.77), followed by SIRI (~0.72) and PLR (~0.70), with NLR and SII modest but significant. Optimal cut-offs (Youden’s *J*) and operating characteristics are reported in **Table 2** and are referenced in the legend.

**Figure 3:**
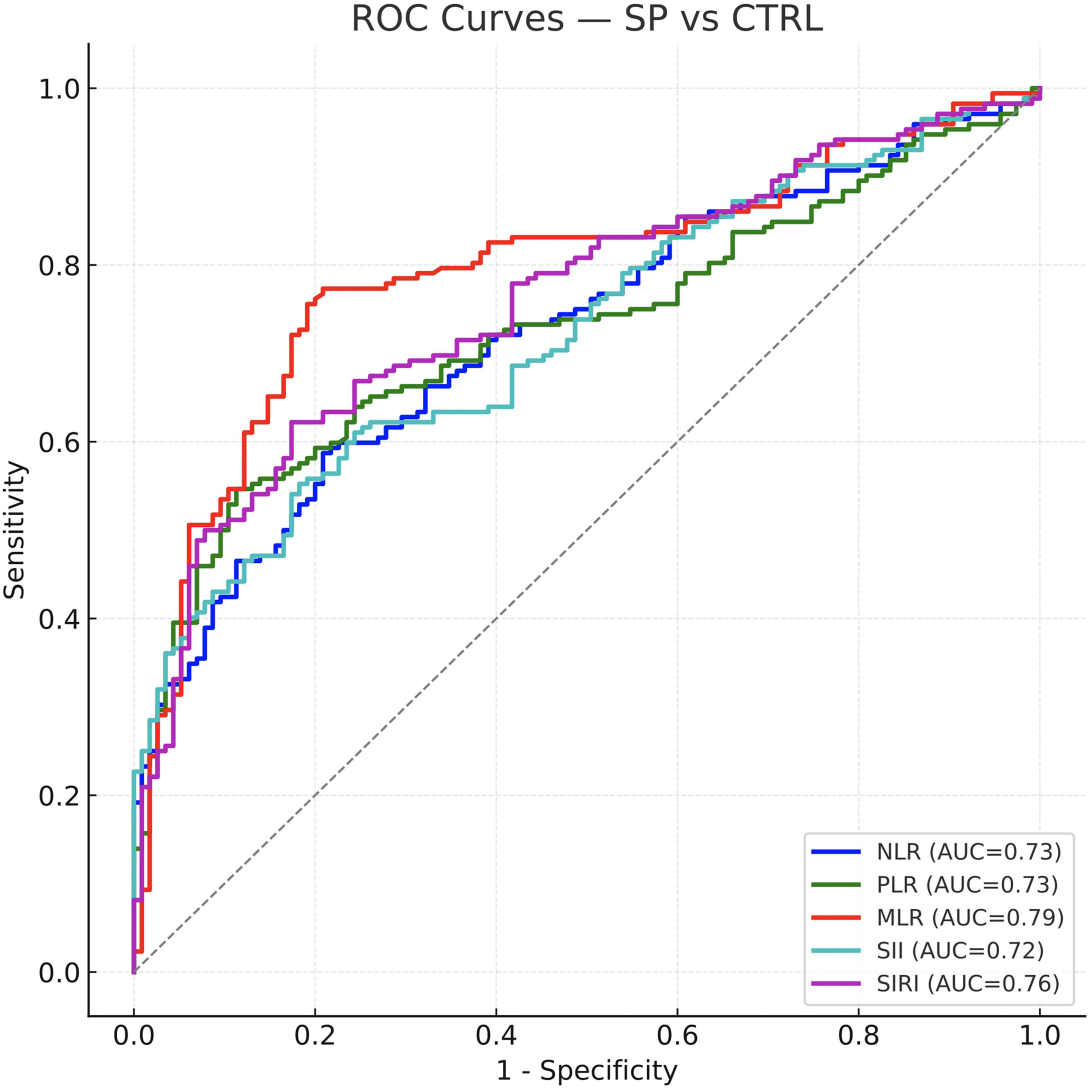
ROC curves for diagnosing seropositive RA (SP-RA) versus controls. In the seropositive subset, diagnostic accuracy remains high across indices (AUC ~0.72–0.79; all *P* < 0.001). MLR (≈ 0.79) and SIRI (≈ 0.76) perform best, mirroring the overall cohort and underscoring their robustness when RF/ACPA are present. Numerical AUCs and cut-offs are listed in **Table 2**.

**Figure 4:**
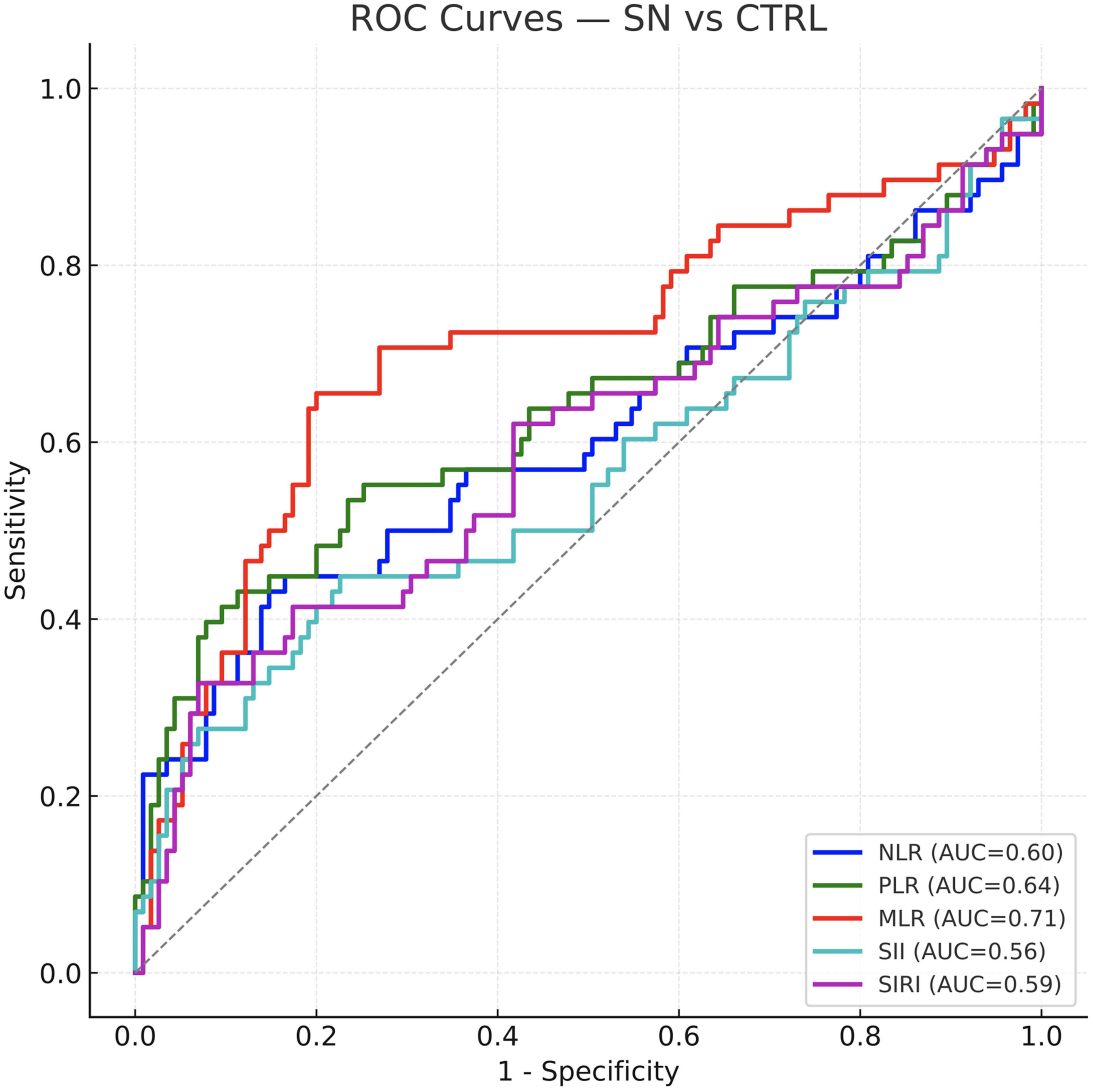
ROC curves for diagnosing seronegative RA (SN-RA) versus controls. Diagnostic performance declines in seronegative disease: MLR retains fair accuracy (AUC ≈ 0.71; *P* < 0.001), NLR/PLR are modest (≈ 0.60–0.64), while SII (~0.56; *P* = 0.09) and SIRI (~0.59) contribute little. These curves visualize the attenuation seen in **Table 2** and highlight the unmet need for reliable markers in SN-RA.

In seropositive RA (SPRA), findings mirrored the overall cohort: AUCs ranged 0.72–0.79, with MLR highest (AUC = 0.793) and SIRI also strong (AUC = 0.759); all indices significantly distinguished SPRA from controls (P < 0.001). In seronegative RA (SNRA), discrimination was attenuated. Only MLR retained fair performance (AUC = 0.707, P < 0.001). NLR and PLR were modest (AUCs ~0.60–0.64), while SII had little diagnostic utility (AUC = 0.56, P = 0.09) and SIRI was similarly weak (AUC = 0.59, P = 0.02). ROC curves for the combined cohort and the two serostatus subgroups are shown in **Figures 2**–**4**; corresponding statistics appear in **Table 2**.

### 3.5 Correlations between indices and disease activity

Within the RA cohort, NLR, SII, and SIRI correlated positively (though moderately) with acute-phase reactants and composite activity scores. SII showed one of the strongest associations with CRP (ρ ≈ 0.39) and DAS28-ESR (ρ ≈ 0.32), with similar magnitudes for SIRI and NLR (all P < 0.001). Correlations with CDAI were weaker but remained significant for NLR, SII, and SIRI. In contrast, PLR showed little relationship to clinical activity, and MLR exhibited only limited associations. Full coefficients are provided in **Table 3**, and serostatus-stratified correlation results are summarized in **Table 4**.

**Table 3:** Spearman correlation coefficients (ρ) between inflammatory indices and RA disease activity measures.

Index	ESR (ρ)	CRP (ρ)	DAS28-ESR (ρ)	DAS28-CRP (ρ)	SDAI (ρ)	CDAI (ρ)
NLR	0.258***	0.321***	0.276***	0.331***	0.255***	0.174**
PLR	0.168*	0.184**	0.098 (ns)	0.114 (ns)	0.049 (ns)	-0.008 (ns)
MLR	0.130*	0.256***	0.135*	0.213**	0.140*	0.083 (ns)
SII	0.322***	0.389***	0.317***	0.377***	0.276***	0.175**
SIRI	0.273***	0.392***	0.308***	0.399***	0.309***	0.211**

*Significance codes:* ***P < 0.001; **P < 0.01; *P < 0.05; ns: not significant.

Table content listing correlation ρ values of NLR, PLR, MLR, SII, SIRI with ESR, CRP, DAS28-ESR, DAS28-ESR, CDAI, SDAI, including significance codes.

ESR, erythrocyte sedimentation rate; CRP, C-reactive protein; DAS28-ESR, Disease Activity Score-28 joints with ESR; DAS28-CRP, Disease Activity Score-28 joints with CRP; CDAI, Clinical Disease Activity Index; SDAI, Simplified Disease Activity Index.

**Table 4:** Correlations between CBC-derived inflammatory indices and disease activity measures stratified by serostatus.

Index	ESR		CRP		DAS28-ESR		DAS28-CRP		SDAI		CDAI	
	ρ	P	ρ	P	ρ	P	ρ	P	ρ	P	ρ	P
SPRA (n = 172)												
NLR	0.202	0.008	0.302	<0.001	0.231	0.002	0.301	<0.001	0.236	0.002	0.148	0.054
PLR	0.128	0.096	0.21	0.006	0.081	0.294	0.118	0.125	0.058	0.455	−0.041	0.596
MLR	0.111	0.151	0.259	<0.001	0.108	0.16	0.192	0.012	0.108	0.161	0.052	0.503
SII	0.303	<0.001	0.4	<0.001	0.309	<0.001	0.383	<0.001	0.285	<0.001	0.161	0.036
SIRI	0.252	<0.001	0.389	<0.001	0.281	<0.001	0.381	<0.001	0.282	<0.001	0.188	0.015
SNRA (n = 58)												
NLR	0.36	0.005	0.341	0.009	0.336	0.01	0.33	0.011	0.22	0.097	0.29	0.027
PLR	0.25	0.059	0.061	0.65	0.075	0.578	0.005	0.972	−0.078	0.559	0.102	0.445
MLR	0.101	0.45	0.173	0.195	0.098	0.464	0.122	0.363	0.081	0.543	0.191	0.151
SII	0.34	0.009	0.345	0.008	0.268	0.042	0.271	0.04	0.162	0.223	0.288	0.029
SIRI	0.268	0.042	0.412	0.001	0.323	0.014	0.381	0.003	0.315	0.016	0.364	0.005

Spearman’s rank correlation coefficients (ρ) and two-sided *P* values are shown for associations between CBC-derived inflammatory indices (NLR, PLR, MLR, SII, and SIRI) and inflammatory markers or composite disease activity measures in rheumatoid arthritis, stratified by serostatus. SPRA denotes seropositive rheumatoid arthritis (n = 172) and SNRA seronegative rheumatoid arthritis (n = 58).

### 3.6 Discrimination of active versus non-active disease

**Figure 5:**
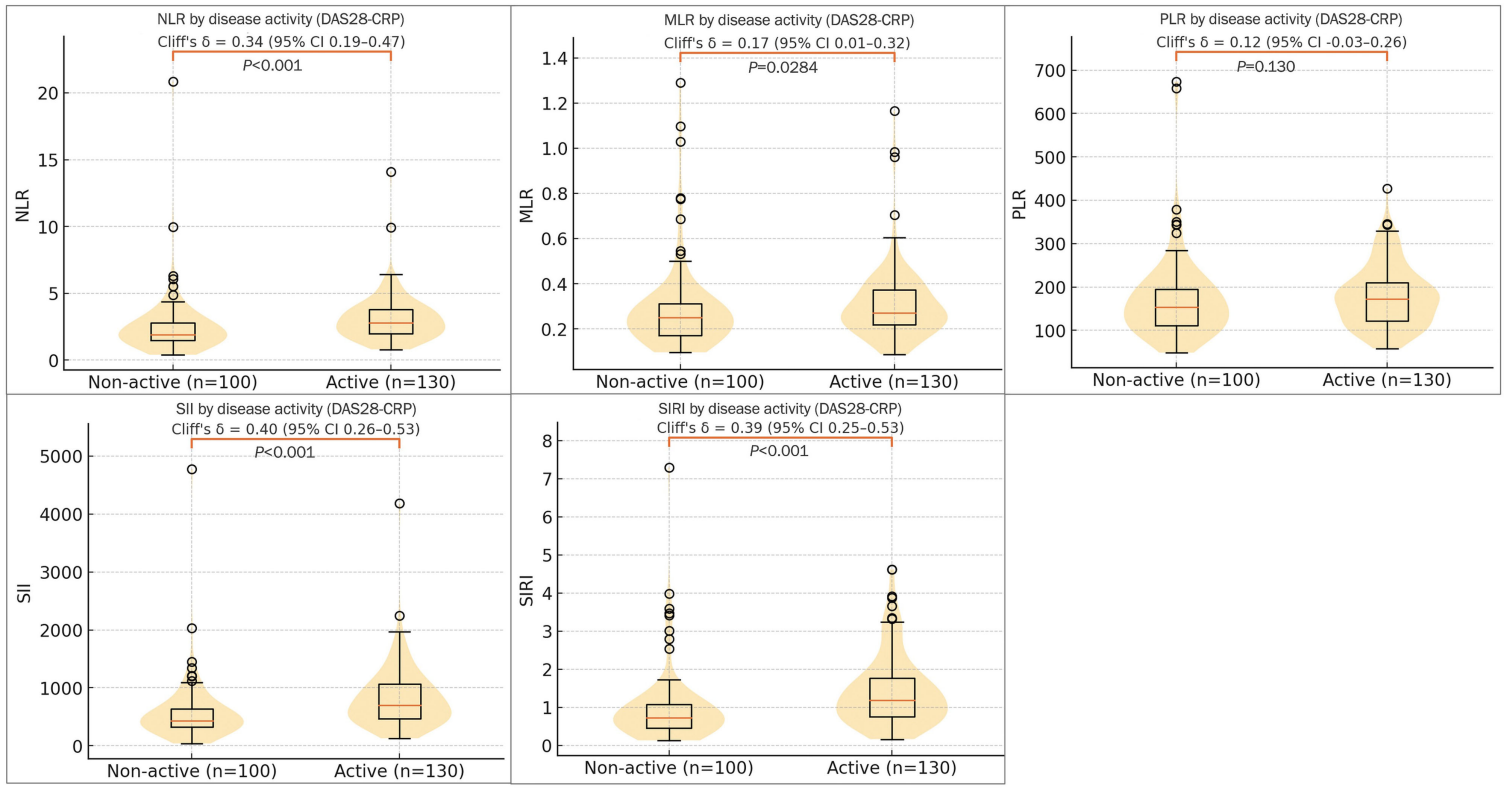
Distributions of CBC-derived indices by RA disease activity (DAS28-CRP). NLR, MLR, PLR, SII, SIRI. Non-active = remission/low; Active = moderate/high (n = 100 vs 130). Violin+box plots show higher NLR, MLR, SII, SIRI in the Active group (Mann–Whitney U; *P* values and Cliff’s δ with 95% CI annotated), whereas PLR shows no significant difference. These distributional contrasts align with activity-ROC results (**Table 5**), in which SII/SIRI provide the highest—though moderate—AUCs for identifying active disease.

When classifying RA activity as moderate/high versus remission/low, all indices except PLR were significantly higher in the active disease group (**Figure 5**). Using DAS28-CRP, SII achieved the highest AUC for active disease (AUC = 0.700), followed by SIRI (AUC = 0.695) and NLR (AUC = 0.668); MLR was lower (AUC = 0.584). With SDAI, SIRI and SII again performed best (AUCs = 0.662 and 0.652), with NLR at 0.626 and MLR not reaching significance (AUC = 0.548, P = 0.058). **Table 5** provides cut-offs and performance (including 95% CIs) for both activity definitions.

**Table 5:** ROC analysis of CBC-derived inflammatory indices for identifying moderate-to-high disease activity in RA.

Activity definition	Cut-off (95% CI)	AUC (95% CI)	Sensitivity % (95% CI)	Specificity % (95% CI)	P-value
DAS28-CRP (moderate/high vs low)					
NLR	2.507 (1.902–3.356)	0.668 (0.599–0.738)	61.5 (53.0–69.5)	69.0 (59.4–77.2)	<0.001
MLR	0.343 (0.171–0.354)	0.584 (0.519–0.651)	33.8 (26.3–42.3)	86.0 (77.9–91.5)	0.006
SII	599.2 (418.7–802.5)	0.700 (0.627–0.767)	60.8 (52.2–68.7)	74.0 (64.6–81.6)	<0.001
SIRI	1.393 (0.877–1.402)	0.695 (0.623–0.764)	46.2 (37.8–54.7)	88.0 (80.2–93.0)	<0.001
SDAI (moderate/high vs low)					
NLR	2.411 (1.903–3.792)	0.626 (0.550–0.702)	65.3 (54.1–75.1)	56.8 (48.9–64.3)	<0.001
MLR	0.343 (0.214–0.364)	0.548 (0.476–0.620)	36.0 (26.1–47.3)	80.0 (73.0–85.5)	0.058
SII	565.6 (443.6–1002.1)	0.652 (0.573–0.726)	69.3 (58.2–78.6)	56.8 (48.9–64.3)	<0.001
SIRI	1.393 (0.752–1.435)	0.662 (0.584–0.738)	52.0 (40.9–62.9)	78.7 (71.6–84.4)	<0.001

ROC curve analyses were conducted to assess the ability of CBC-derived inflammatory indices to discriminate active rheumatoid arthritis (moderate or high disease activity) from remission or low disease activity, using DAS28-CRP and SDAI as activity definitions. Optimal cut-off values were selected by the Youden index. AUCs are reported with 95% confidence intervals (CIs), and sensitivity and specificity are presented with corresponding 95% CIs. The 95% CIs for cut-off values were obtained by nonparametric bootstrap resampling (2,000 replicates). PLR was not included in this table because no significant difference was observed between activity groups.

### 3.7 Focused analysis of the seronegative subset

In the 58 RF/ACPA double-negative patients compared with 115 controls, median NLR, PLR, MLR, and SIRI were all higher in SNRA than in controls (all P < 0.05), whereas SII did not differ (P = 0.179). ROC analysis identified MLR as the best single discriminator in SNRA (AUC = 0.707). These results are summarized in **Table 6** alongside the corresponding ROC panel for the seronegative subset.

**Table 6:** Diagnostic performance of CBC-derived inflammatory indices in seronegative rheumatoid arthritis (SNRA) versus healthy controls.

Index	RA group (IQR)	Control (IQR)	P	AUC (95% CI)	Cut-off (95% CI)	Sensitivity % (95% CI)	Specificity % (95% CI)
NLR	2.10 (1.40–3.13)	1.75 (1.43–2.24)	0.026	0.604 (0.505–0.699)	2.55 (1.92–3.58)	43.1 (31.2–55.9)	85.2 (77.6–90.6)
PLR	148.3 (108.4–197.2)	121.5 (103.1–143.1)	0.003	0.637 (0.534–0.731)	175.8 (143.3–187.1)	39.7 (28.1–52.5)	92.2 (85.8–95.8)
MLR	0.24 (0.17–0.29)	0.18 (0.15–0.20)	<0.0001	0.707 (0.614–0.797)	0.21 (0.20–0.23)	65.5 (52.7–76.4)	80.0 (71.8–86.3)
SII	444 (307–741)	421 (297–518)	0.179	0.563 (0.462–0.661)	543 (543–854.9)	44.8 (32.7–57.5)	77.4 (68.9–84.1)
SIRI	0.72 (0.46–1.18)	0.58 (0.42–0.78)	0.044	0.594 (0.496–0.693)	1.07 (0.60–1.18)	32.8 (22.1–45.6)	93.0 (86.9–96.4)

AUC values are presented with 95% confidence intervals (CIs). Sensitivity, specificity, and cut-off values are reported with corresponding 95% CIs.

### 3.8 Summary of key findings

Taken together, MLR emerged as the most informative marker for differentiating RA from healthy controls across serostatus, whereas SIRI—along with SII and NLR—tracked most closely with inflammatory activity and provided the highest (moderate) AUCs for identifying active disease. PLR consistently underperformed in both diagnostic and activity-related contexts. Importantly, all indices lost accuracy in seronegative RA, underscoring the need for better biomarkers in this clinically challenging subgroup.

## 4 Discussion

Consistent with prior cohorts and meta-analyses, all five CBC-derived indices were elevated in RA versus controls, supporting their role as hematologic proxies of systemic inflammation. For example, Jin et al. showed that both NLR and PLR were markedly higher in RA than in non-RA groups (26), and meta-analysis likewise found higher NLR and PLR in RA versus controls (27, 28). These increases are biologically plausible given RA’s immune dysregulation, in which neutrophils and platelets are activated while lymphocyte counts decline, reflecting chronic systemic inflammation (12). In line with earlier work, we also observed positive correlations between NLR and disease activity markers (e.g., CRP, DAS28); similar associations have been reported in cohort studies and meta-analyses (29, 30). Moreover, several studies demonstrated higher NLR (and sometimes PLR) in active RA than in remission (31, 32).

To our knowledge, few studies have assessed all five indices in parallel within a single RA cohort; our analysis provides such head-to-head evidence and clarifies their comparative diagnostic and activity-related performance; most assessed one or two (e.g., NLR/PLR, or SIRI alone) (14, 33, 34). By examining them side-by-side, we showed that each index distinguished RA from controls (all *P* < 0.001 in our dataset).

Another contribution of this study is the explicit evaluation of seronegative RA (SN-RA). Prior literature often under-reports serostatus or focuses on seropositive disease (3). We found that SN-RA patients also exhibit elevated NLR, SII, and SIRI versus healthy controls, with levels broadly comparable to seropositive RA in our cohort. This aligns with data indicating that several CBC-derived indices do not differ materially by RF/ACPA status (33) and underscores their potential serology-independent utility. Given the diagnostic challenges of SN-RA, especially when traditional antibodies are absent, our findings support incorporating these indices as adjunct markers (3).

Despite overall agreement with prior work, two observations diverged from expectations. First, the PLR showed weaker diagnostic and activity-related performance in our cohort. Although higher in RA than controls, PLR contributed less to multivariable models and correlated only modestly with activity. Our results mirror a large cross-sectional analysis showing modest sensitivity (~57%) of PLR for active disease, underscoring its limited individual-level utility (35). Platelet counts are susceptible to confounding (iron deficiency, glucocorticoids, comorbidities), and antirheumatic therapy can dampen thrombocytosis, potentially lowering PLR’s signal-to-noise.

Second, the MLR displayed an attenuated association with RA activity. Although monocytes are central to synovial macrophage biology, we observed minimal differentiation of MLR across activity states and weak correlation with DAS28-CRP relative to NLR/SII/SIRI. This mirrors findings from Medicine (2021), where LMR (the inverse of MLR) did not differ significantly between active and remission groups and was not a useful independent activity marker (35). A plausible explanation is that peripheral monocyte counts fluctuate less with acute disease shifts (due to tissue trafficking or plateauing), yielding a narrower dynamic range for activity monitoring. By contrast, neutrophil-centric measures (NLR, SII, SIRI) may more promptly reflect cytokine-driven inflammatory surges.

Elevations in NLR, PLR, SII, and SIRI reinforce a model in which innate immune activation (neutrophils/platelets/monocytes) coexists with relative lymphopenia in RA, driven by cytokine networks (e.g., TNF-α, IL-6) and sustained synovial inflammation (36–38). In particular, neutrophils and monocytes are implicated in synovial damage and systemic inflammation; thus, higher NLR and SIRI may index neutrophil/monocyte expansion and activation (36, 39). Conceptually, SII (platelets × neutrophils / lymphocytes) integrates pro-inflammatory myeloid responses with reduced lymphocyte-mediated regulation, offering a composite snapshot of systemic immune-inflammation (33).

In terms of clinical application, CBC-derived indices may serve as adjunctive tools to support early detection or triage, rather than as stand-alone diagnostic markers. In primary care settings, elevated NLR or SII values in patients presenting with inflammatory joint symptoms may raise suspicion for inflammatory arthritis while awaiting definitive serological testing (e.g., RF/ACPA) or imaging confirmation. Pooled evidence suggests acceptable diagnostic accuracy for NLR (AUC ≈ 0.70–0.76 for RA presence or active disease) and moderate accuracy for PLR in distinguishing RA from non-RA conditions, albeit with substantial heterogeneity across studies (28).

Beyond initial assessment, these indices may contribute to the monitoring of established RA as complements to clinical evaluation and conventional acute-phase reactants. Notably, imaging studies have shown that ultrasound can detect subclinical synovitis in patients classified as being in remission by DAS28, highlighting limitations of standard clinical and laboratory markers; in this context, modestly elevated NLR or SII values may reflect residual systemic inflammation and warrant closer follow-up rather than immediate treatment escalation (38, 40).

Finally, accumulating population-based evidence has linked NLR, SII, and SIRI to adverse long-term outcomes in RA, including all-cause and cardiovascular mortality as well as skeletal complications such as vertebral fractures (13, 39, 41, 42). These findings suggest potential prognostic relevance of CBC-derived indices, although their interpretation should remain cautious given their non-specific nature and susceptibility to treatment- and comorbidity-related confounding.

This study has three main limitations. First, CBC-derived inflammatory indices are non-specific markers of systemic inflammation and therefore cannot substitute for established diagnostic tools such as RF/ACPA testing, imaging modalities, or comprehensive clinical assessment; their potential value lies primarily in an adjunctive role. In addition, this was a single-center, retrospective, cross-sectional analysis, which limits causal inference and generalizability.

Second, the comparison was restricted to RA versus healthy controls, preventing assessment of test specificity against other inflammatory arthritides, osteoarthritis, or infection. CBC-derived indices may also be influenced by medications, comorbid conditions, smoking status, or iron deficiency. Moreover, certain RA therapies (e.g., IL-6 receptor blockade and JAK inhibitors) can alter leukocyte and platelet profiles as well as acute-phase reactants, potentially confounding CBC-derived indices and their correlations with disease activity.

Third, we lacked longitudinal sampling and outcome follow-up, precluding evaluation of dynamic changes, treatment responsiveness, flare prediction, or long-term prognostic performance. The cut-off values and AUC estimates reported here were derived within this dataset and have not yet been externally validated. Consequently, our findings should be considered exploratory and hypothesis-generating, pending confirmation in prospective, multicenter studies.

Future research should focus on prospective validation and on mechanistic work linking these hematologic indices to cytokine pathways and synovial immune phenotypes.

## 5 Conclusion

In conclusion, our study provides a comprehensive comparative evaluation of five readily available blood-derived inflammatory markers in RA. We demonstrated that NLR, PLR, MLR, SII, and SIRI are all elevated in RA patients relative to healthy individuals, reflecting the heightened innate immune activation characteristic of this disease. Among these indices, those incorporating neutrophils and platelets (NLR, SII, SIRI) showed the strongest associations with RA diagnosis and disease activity, whereas PLR and MLR appeared less responsive to disease fluctuations. Particularly MLR remained informative in seronegative RA, whereas SII showed limited diagnostic utility. Taken together, these inexpensive indices have potential clinical utility as adjuncts for early detection of RA and for monitoring inflammation and disease activity over time.

## Data Availability

The original contributions presented in the study are included in the article/supplementary material. Further inquiries can be directed to the corresponding authors.
